# Minimal vertex model explains how the amnioserosa avoids fluidization during *Drosophila* dorsal closure

**Published:** 2023-12-20

**Authors:** Indrajit Tah, Daniel Haertter, Janice M. Crawford, Daniel P. Kiehart, Christoph F. Schmidt, Andrea J. Liu

**Affiliations:** a Speciality Glass Division, CSIR-Central Glass and Ceramic Research Institute, Kolkata, India;; b Department of Physics and Astronomy, University of Pennsylvania, PA, USA;; c Institute of Pharmacology and Toxicology, University Medical Center and Campus Institute Data Science (CIDAS), University of Göttingen, Germany;; d Department of Physics and Soft Matter Center, Duke University, Durham, NC, USA;; e Department of Biology, Duke University, Durham, NC, USA

**Keywords:** *Drosophila* dorsal closure, morphogenesis, epithelial tissue, amnioserosa, vertex model, tissue rigidity

## Abstract

Dorsal closure is a process that occurs during embryogenesis of *Drosophila melanogaster*. During dorsal closure, the amnioserosa (AS), a one-cell thick epithelial tissue that fills the dorsal opening, shrinks as the lateral epidermis sheets converge and eventually merge. During this process, the aspect ratio of amnioserosa cells increases markedly. The standard 2-dimensional vertex model, which successfully describes tissue sheet mechanics in multiple contexts, would in this case predict that the tissue should fluidize via cell neighbor changes. Surprisingly, however, the amnioserosa remains an elastic solid with no such events. We here present a minimal extension to the vertex model that explains how the amnioserosa can achieve this unexpected behavior. We show that continuous shrink-age of the preferred cell perimeter and cell perimeter polydispersity lead to the retention of the solid state of the amnioserosa. Our model accurately captures measured cell shape and orientation changes and predicts non-monotonic junction tension that we confirm with laser ablation experiments.

The developmental stage of dorsal closure in *Drosophila melangaster* occurs roughly midway through embryogenesis and provides a model for cell sheet morphogenesis (1–4). The amnioserosa (AS) consists of a single sheet of cells that fills a gap on the dorsal side of the embryo separating two lateral epidermal cell sheets. During closure, the AS shrinks in total area, driven by non-muscle myosin II acting on arrays of actin filaments in both the AS and actomyosin-rich cables in the leading edge of the lateral epidermis (5–8). Ultimately, the AS disappears altogether. The entire closure process is choreographed by a developmental program that mediates changes in AS cell shapes as well as forces on adherens junctions between cells (9, 10).

One might naively expect cells in the AS, which are glued to their neighbors by molecules such as E-cadherin, to maintain their neighbors, so that the tissue behaves like a soft, elastic solid even as it is strongly deformed by the forces driving dorsal closure. However, the time scale for making and breaking molecular bonds between cells (ms) is far faster than the time scale for dorsal closure (hours). As a result, cells can potentially slip past each other while maintaining overall tissue cohesion. Such neighbor changes could cause epithelial tissue to behave as a viscous fluid on long time scales rather than an elastic solid, as it does during convergent extension (11). Vertex models (12–21) have provided a useful framework for how tissues can switch between solid and fluid behavior (16, 17), and have had remarkable success in describing experimental results (11, 22–25). These models make the central assumption that internal forces within a tissue are approximately balanced on time scales intermediate between ms and hours, and have successfully described phenomena such as pattern formation, cell dynamics, and cell movement during tissue development (26). Force balance is captured by minimizing an energy that depends on cell shapes. In the standard vertex model, energy barriers are lower when cells have high aspect ratios, so higher/lower cell aspect ratios correspond to fluid/solid behavior.

During dorsal closure, significant changes in AS cell shapes are observed. According to the standard vertex model, the observed high values of mean cell shape aspect ratio should render the tissue fluid (15–17). Nonetheless, there is considerable experimental evidence that the AS remains *solid* during dorsal closure with *no* neighbor exchanges (27–29). We have examined individual junction lengths using live embryo imaging in an extensive data set comprising 10s of embryos, each with 100s of cells and, in agreement with the literature, did not find any vanishing junctions, and hence any neighbor exchanges, except when cells left the AS (cell ingression).

Vertex models might simply fail to describe tissue mechanics at this stage of development. The success of vertex models in describing many other tissues, however, begs the question: can the models be tweaked to capture tissue mechanics of the AS during dorsal closure, and could this point to an important physiological control mechanism? Here we introduce a minimal extension to the standard vertex model that quantitatively captures results from comprehensive experimental datasets obtained from time-lapse microscopy recordings.

## 1. Modeling and experimental analysis

**Fig. 1.**
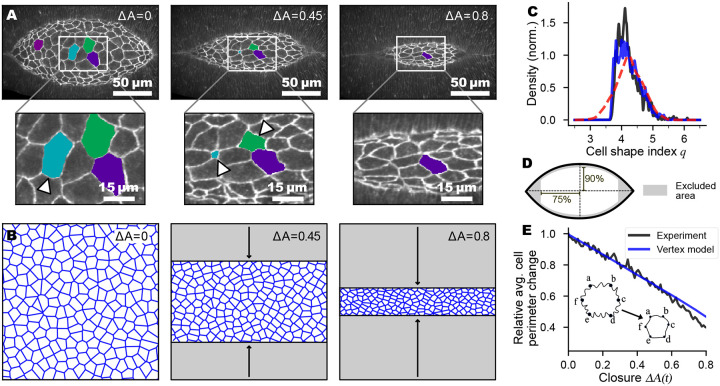
Experiment and vertex model for dorsal closure. (**A**) The geometry of the dorsal hole during early (left), middle (center), and late (right) dorsal closure. Enlargements show tissue with selected cells, several of which ingress (highlighted by triangles). (**B**) We model the dorsal closure process as a quasistatic uni-axial deformation. The geometry of the model is shown at the beginning (left), in the middle (center, at 45% closure), and towards the end (right, 80% closure) of the process. $\Delta A\left(t\right)=\frac{A_{0}-A\left(t\right)}{A_{0}}$ is the fractional change in total AS area of the closure process, where $A_{0}$ is the AS area at the onset of dorsal closure. (**C**) An initial normal distribution of the preferred shape index of the model tissue (dashed red) with the standard deviation adjusted to be 0.45, leads to a distribution of the actual shape index after minimization (solid blue) that is in excellent agreement with the distribution of the experimentally observed shape index (solid black) at the beginning of dorsal closure. (**D**) Sketch of AS tissue regions included in model comparison (white center), with edge regions excluded (gray regions). (**E**) In the model, we reduce the preferred cell perimeter at a linear rate (blue) to capture the experimentally observed decrease of junction lengths (black). For comparison, we normalize the average perimeter by its value at the onset of the process. Inset: schematic representation of the reduction of cellular junction length and apical area during dorsal closure.

Our starting point is a standard two-dimensional cellular vertex model (12–21, 30, 31) (short introduction to vertex models in SI section A). The AS is represented as a single-layer sheet of polygonal cells that tile the entire area, as described below. In our model, we approximate the shape of the AS tissue (Fig. 1A) with a rectangle whose long axis corresponds to the anterior-posterior axis of the embryo (Fig. 1B, details see SI section B). During simulated dorsal closure, the positions of the vertices are continually adjusted to maintain the mechanical energy of the tissue at a minimum, or equivalently, to balance the forces exerted on each vertex. The mechanical energy of the standard vertex model is defined as

[1] $$E=\sum_{i=1}^{N}\frac{1}{2}k_{a}{\left(a_{i}-a_{0}\right)}^{2}+\frac{1}{2}k_{p}{\left(p_{i}-p_{0,i}\right)}^{2},$$

where N is the total number of cells, $p_{i}$ and $a_{i}$ are the actual cell perimeters and areas, $p_{0,i}$ and $a_{0}$ are the preferred cell perimeters and area, and $k_{p}$ and $k_{a}$ represent the perimeter and area elastic moduli of the cells, respectively. The first term penalizes apical area changes away from a preferred value, and can arise from cell height changes as well as active contractions in the medio-apical actin network at constant or near constant volume. The second term combines the effects of actomyosin cortex contractility with cell-cell adhesion, where $p_{0,i}$ is the effective preferred cell perimeter (16). For simplicity, we chose $k_{a}=k_{p}=1$ for all cells.

We used time-lapse confocal microscopy to image the entire dorsal closure process in E-cadherin-GFP embryos. We then used our custom machine-learning-based cell segmentation and tracking algorithm to create time series of cell centroid position, area, perimeter, aspect ratio, and individual junction contour lengths for every cell in the AS (32). At the onset of closure we find that cells in the AS exhibit considerable variability of the cell shape index $q_{i}=p_{i}/{\sqrt{a}}_{i}$ (Fig. 1C). In the model, we therefore introduce initial polydispersity in the cell shape index through a normal distribution of preferred cell perimeters $p_{0,i}$. We fix the preferred cell area and use it to set our units so that $a_{0}=1$ for all cells, following Ref. (33). The distribution of actual shape index $q_{i}$ after minimizing the mechanical energy in the model is in excellent agreement with the experiments (Fig. 1C).

During a substantial part of closure (Fig. 1A), the leading edges of the two flanking epithelial sheets approach the dorsal mid-line at a roughly constant rate (7). To mimic these dynamics, we linearly decreased the vertical height of the rectangle representing the AS (Fig. 1B) by 0.125 % of the initial height at every step while holding the width fixed. We enforce force balance, minimizing the mechanical energy after each deformation step. We used periodic boundary conditions throughout.

Since closure rates varied from embryo to embryo, we measured progress during closure not in terms of *time*, but in terms of fractional change of the total area of the exposed AS (i.e. the dorsal opening), $\Delta A\left(t\right)=\frac{A_{0}-A\left(t\right)}{A_{0}}$, where $A_{0}$ is a reference area of the AS early during closure. In many prior studies (34, 35), the height of the AS has been used as a descriptor of closure progress. In Fig. S1 we demonstrate that both height and area of the AS decreased monotonically and approximately linearly with time, validating our use of ΔA(t) to mark the progression of closure. We began the analysis of each embryo at $A_{0}=11,000\;\mu {\text{m}}^{2}$, so that we could average over multiple embryos. To exclude complex tissue boundary effects, we excluded cells at the AS borders and the regions at the canthi in the comparison between model and experiment (Fig. 1D).

AS cells reduce their perimeter (inset Fig. 1E) (36) during the closure process by removing a portion of junction material and membranes through endocytosis, while maintaining junction integrity (37, 38). The average perimeter shrinks at a constant rate in the experiments (Fig. 1E). We therefore assume in the model that the *preferred* perimeter $p_{0,i}$ of each cell decreases linearly with ΔA(t) at the same rate (details see SI section B). Note that we do not change the preferred area per cell, $a_{0}=1$. For a more realistic model we could change the preferred area in proportion to the total area of the AS as it shrinks, but that would change only the pressure, and would have no effect on the rigidity transition (39–41).

During dorsal closure, ~ 10% of AS cells ingress into the interior of the embryo (3, 42, 43) (additional cells ingress at the canthi and adjacent to the lateral epidermis). In the model, we removed cells randomly at the experimentally measured rate (see details in SI) so that roughly 10% of the AS cells disappeared over the course of dorsal closure.

For further details of the model and the experiments, see Materials and Methods and Supplemental Information.

## 2. Results

We tracked the following quantities during dorsal closure, in model and experiments: mean cell shape index $\bar{q}=\langle p_{i}/\sqrt{a_{i}}\rangle$, mean aspect ratio $\bar{\alpha }$ (see SI section D), orientational order parameter $\bar{Q}=\langle \text{cos}\left(2\theta \right)\rangle$ (44) (see SI section E) characterizing the degree of cellular alignment (where θ is the angle between the major axis of each cell and the anterior-posterior axis, $\bar{Q}=0$ for randomly aligned cells and $\bar{Q}=1$ for cells perfectly aligned with the AP axis), standard deviation of cell shape index $\sigma_{q}$ and standard deviation of the aspect ratio $\sigma_{\alpha }$.

**Fig. 2.**
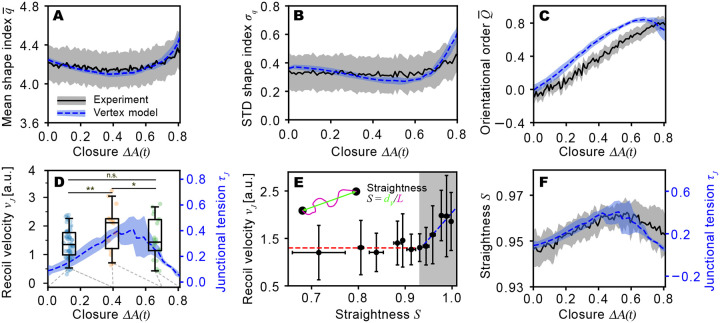
Results from experiment (black solid) and model (blue dashed). (**A**) A comparison of average shape index $\bar{q}$ as a function of $\Delta A\left(t\right)=\frac{A_{0}-A\left(t\right)}{A_{0}}$. Here $A_{0}$ is the AS area at the onset of dorsal closure and A(t) is the area as it shrinks during dorsal closure, so that ΔA(t)=0 at onset. Lines show the mean, and shaded regions indicate the standard deviation among 12 embryos (experiment) or 10 different initial configurations (model). (**B**) Comparison of cell to cell standard deviation of the shape index $\left(\sigma_{q}\right)$ during dorsal closure. (**C**) Orientational order parameter $\left(\bar{Q}\right)$ of the cells during dorsal closure. (**D**) Experimental initial junction recoil velocity (left y-axis) of the vertices after performing laser ablation of the junction, and predicted average cellular cortical tension $\left({\bar{\tau }}_{J}\right)$ (right y-axis) of the model during dorsal closure. The boxplots represent data across three intervals of ΔA (ΔA<0.4, 0.4≤ΔA<0.7, ΔA≥0.7). Whiskers extend to the 5th and 95th percentiles, while the boxes delineate the interquartile range, and the horizontal lines within the boxes indicate the median values. An ANOVA followed by a post-hoc Tukey’s HSD test was conducted to assess statistical significance (*: p<0.1, **: p<0.05). We performed and evaluated cuts of N=97 junctions. (**E**) Average initial recoil velocity of vertices after laser cutting as a function of junction straightness (ratio of the inter-vertex distance $\left(d_{v}\right)$ to the junction length (L), see inset) immediately before cutting. Junction recoil velocity is independent of junction straightness (fitted with the red dashed line) until $S=d_{v}/L≳0.93$. The crossover point at $d_{v}/L\approx 0.93$ marks the intersection of the red and blue dashed lines; the latter fits the data points in the gray-shaded region, indicating that the recoil velocity increases strongly and approximately linearly with junction straightness in this regime. (**F**) Comparison of experimental junction straightness (left y-axis) and model cellular junction tension (right y-axis) during dorsal closure.

We compare experimental data and simulations without any parameter modifications, adjustments, or rescaling with time. Considering the simplicity of the model, the agreement is remarkably good, both for cell shape and cell shape variability (Fig. 2A,B) as well as cellular alignment (Fig. 2C). The mean and standard deviation of cell aspect ratio agree equally well (SI F and Fig. S2). As expected, the error bars (shaded region) in the experimental data, which represent variations between different embryos, are significantly larger than those in the simulation data, which represent only variations between initial configurations based on a single distribution of cell shape indices from the distribution measured over all embryos (Fig. 1C). In the experiments, there is intrinsic embryo-to-embryo variability that we did not include in our model for simplicity.

In experiment and model, the mean shape index $\bar{q}$ initially decreases, reaches a minimum at ΔA≈0.55 and then increases (Fig. 2A). In the model, this behavior arises from two competing effects. (i) Decreasing preferred mean perimeter (Fig. 1E) implies a decreasing preferred mean shape index, ${\bar{q}}_{0}=\langle p_{0,i}/\sqrt{a_{0,i}}\rangle$. According to Eq. 1, this tends to drag down $\bar{q}$, causing the decrease up to ΔA≈0.55. (ii) As dorsal closure progresses, the overall shape of the tissue becomes more and more anisotropic (Fig. 1A,B), increasing $\bar{q}$. This effect eventually dominates for ΔA≳0.55. This competition between decreasing ${\bar{q}}_{0}$ and increasing anisotropy is also reflected in the width of the q-distribution, $\sigma_{q}$ (Fig. 2B). In the model, the standard deviation of the preferred shape index, $\sigma_{q,0}$, is fixed, but cell to cell variations of the energy E due to $\sigma_{q,0}$ increase with decreasing ${\bar{q}}_{0}$, leading to a narrowing of the distribution, or a decrease in $\sigma_{q}$. On the other hand, vertical shrinking of the system late in the closure process leads to greater $\sigma_{q}$ (Fig. 2B). The increasing anisotropy during closure leads to greater alignment of cells along the anterior-posterior axis, reflected in an increased orientational order parameter $\bar{Q}$ (Fig. 2C).

A strength of the vertex model is that it predicts not only cell shape and orientation distributions but also mechanical cell-level properties of the AS, including, for example, the cell junction tension $\tau_{J}$, defined as (24, 45)

[2] $$\tau_{J}=k_{p}\left(p_{i}-p_{0,i}\right)+k_{p}\left(p_{j}-p_{0,j}\right),$$

where i, j denote cells that share a given junction. Relative values of junction tension can be estimated experimentally from the initial recoil velocity $v_{r}$ when a junction is severed using laser ablation (14, 46–49). Our model predicts that the average junction tension ${\bar{\tau }}_{J}$ rises until the fractional area of the AS reaches ΔA(t)≈0.55, and then decreases as dorsal closure continues (Fig. 2D). To test this prediction, we conducted laser cutting experiment at different stages of closure. We find that the recoil velocity changed in a non-monotonic manner (Fig. 2D), as the model predicts.

An alternative way to estimate junction tension from imaging data of unperturbed embryos is to analyze the straightness of junctions. A wiggly junction would be expected to be free of tension, whereas a straight junction should support tension. We define junction straightness as $S=d_{v}/L$ (Fig. 2E, inset), where $d_{v}$ is the distance between vertices for a given junction and L is the contour length of the junction. We examined the relation between S and the initial recoil velocity upon cutting, $v_{r}$, and observed that $v_{r}$ is independent of S for S≲0.93 but then rises linearly with increasing S above this threshold (Fig. 2E). It is reasonable to assume that junction straightness S is proportional to the tension $\tau_{J}$ predicted by our model. This is verified in Fig. 2F, showing the same non-monotonicity for both quantities with peaks occurring at ΔA(t)≈0.55.

Why is the junction tension non-monotonic? In vertex models, junction tension and cell stiffness are related to cell shape index (16, 22, 23, 45). According to Eq. 2, junction tension is given by the difference between cell preferred perimeter $p_{0.i}$ and the actual perimeter $p_{i}$ for the two cells sharing a given junction. Below ΔA=0.55, $p_{i}-p_{0,i}$ increases with ΔA, leading to an increase of ${\bar{\tau }}_{J}$. For ΔA≥0.55, $p_{i}-p_{0.i}$ decreases, leading to a decrease of ${\bar{\tau }}_{J}$.

A striking result of the standard vertex model (Eq. 1) is the prediction of a transition from solid to fluid behavior as the average shape index increases above ${\bar{q}}_{c}=3.81$ (16), in excellent agreement with a number of experiments in various epithelial tissue models (22, 50–52). Inspection of Fig. 2A shows that $\bar{q}>3.81$ during the entire process of dorsal closure, suggesting that the AS should be fluid. However, the complete absence of T1 events (cell neighbor changes) shows conclusively that the AS is not fluid but solid.

Which of the extensions of the standard vertex model (Eq. 1) that we have incorporated in our model are responsible for the solid nature of the AS? It is known that cellular shape heterogeneity (33) and orientational ordering (11) both enhance rigidity in vertex models (detailed analysis of orientational alignment see SI section H). In our case, cellular heterogeneity remains essentially constant during dorsal closure, but orientational ordering increases due to uniaxial deformation. Isotropic deformation (SI section I), in contrast, does not lead to orientational order (Fig. S5B), as one might expect. The incorporation of uniaxial deformation is important since trends in $\bar{q}$, $\sigma_{q}$ and junction tension (Fig. S5A,D,C) with closure fail to agree with experimental results if we apply isotropic deformation. However, we find that our model predicts solid behavior even for isotropic deformation, showing that uniaxial deformation is not needed for this aspect. We also find that cell ingression at the levels seen experimentally has almost no effect on the solid behavior in the model. This leaves the progressive decrease of preferred cell perimeter as crucial for maintaining solid response.

For the tissue to behave as a solid, non-zero tension cell junctions must form continuous paths that extend across the entire system in all directions (33, 53)–in other words, they must *percolate*. Percolation requires the fraction of junctions with non-zero tension, $f_{r}$ to be larger than a critical fraction $f_{c}$. The rigidity transition can be driven either by altering $f_{r}$ or $f_{c}$, or both. Note that for random Voronoi tessellations in a square system, $f_{c}\approx 0.66$ (54–56). The topology of such networks is similar to that of the standard vertex model (Eq. 1), so that $f_{c}\approx 0.66$ can be taken as a reasonable approximation. We show in the Supplemental Information section G that $f_{c}$ remains fixed during dorsal closure, despite uniaxial deformation of the AS. Interestingly, the tissue would fluidize without the progressive decrease of preferred cell perimeter (Fig. S3B).

**Fig. 3.**
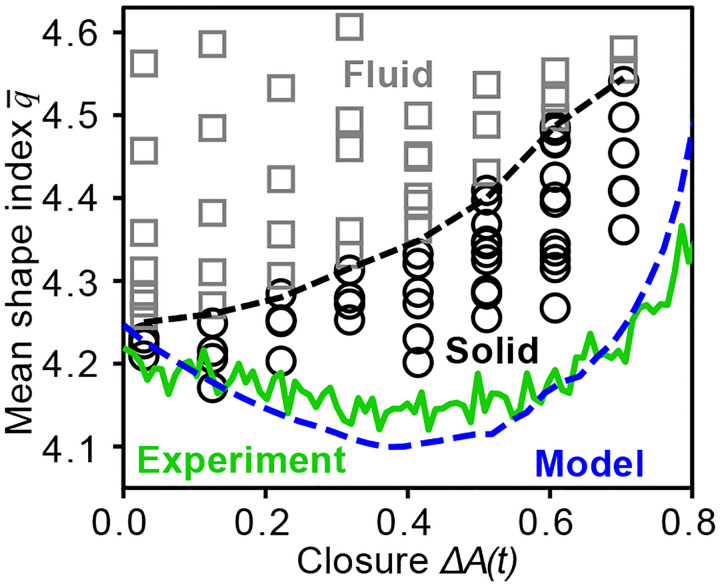
Phase diagram of amnioserosa during dorsal closure. Phase diagram in $\bar{q}$ vs. ΔA space, with solid (open black circles) and fluid (open gray squares) states as shown. The solid-fluid transition is marked by the black dashed line. Both experiment (green dashed line) and model (blue dashed line) are within the solid phase throughout dorsal closure.

Fig. 3 summarizes our results in the form of a phase diagram of our model, obtained by evaluating the fraction of rigid junctions $f_{r}$ at discrete values of mean cell shape index and ΔA. The phase boundary corresponds to the percolation transition of nonzero-tension junctions, $f_{r}=f_{c}\approx 0.66$. The system always remains in the solid phase, consistent with the experimental observations (blue dashed line). As explained earlier, $\bar{q}$ initially decreases because the decreasing preferred perimeter pulls actual cell perimeters down. Eventually, however, the elongation of cells due to uniaxial deformation overcomes this effect, causing $\bar{q}$ to increase.

## 3. Discussion

We find experimentally that the AS remains in a solid phase (*i.e*. with no cell neighbor exchanges) during dorsal closure. One might not be surprised since cells adhere to each other. It is important to realize, though, that cadherins have rapid on-off kinetics and the actin cortex has rapid turnover on the time scale of dorsal closure. As a result, adhesion cannot necessarily prevent cell neighbor switching; it merely guarantees tissue cohesion. Indeed, the standard vertex model predicts that when cells are highly elongated, the barriers to neighbor switching should be low and the tissue should be fluid (11, 17, 22, 23, 57). from the AS cell shapes that barriers should be low and that the AS should be a fluid. Our simple extension of the standard vertex model not only predicts that the tissue should be in the solid phase but also faithfully reproduces a wide range of characteristics of an extensive set of experimental dorsal closure data: cell shape and orientational order, and junction tension, which we inferred passively from image data due to the linear relationship between junction straightness and initial recoil velocity in laser cutting experiments.

Our model achieves this good agreement with only two parameters that are directly derived from experiments. We find that shape polydispersity and active shrinking of the preferred cell perimeters are the two critical factors that enable the tissue to remain solid in spite of extensive cellular and tissue shape changes. These results imply that the solid character of the AS originates from active processes that regulate cell perimeter, including junction complexes and the components of the cell cortex.

This finding raises two questions for future research. First, *how* is the removal of junction material specifically regulated in cells? Second, *why* might it be important for the AS to remain in a solid phase? Perhaps solid behavior during dorsal closure is simply a holdover from the preceding developmental stage of germ band retraction (58). Laser ablation experiments (59) suggest that the AS plays an important assistive role in uncurling of the germ band by exerting anisotropic tension on it. Such anisotropic stress requires the AS to be a solid, not fluid. An interesting future direction for experimental and vertex model studies is to establish whether the AS is solid throughout germ band retraction as well as dorsal closure.

Our results show that vertex models are more broadly applicable than previously thought. Despite the many complex active processes that occur during dorsal closure, we find that only one of them–the active shrinking of a normally-fixed parameter, namely the preferred perimeter–is needed in order to quantitatively describe our experimental observations Similar variation of normally-constant parameters has been shown to allow other systems to develop complex responses not ordinarily observed in passive non-living systems. These include negative Poisson ratios (60, 61) and allostery (62) in mechanical networks, greatly enhanced stability in particle packings (63), and the ability to classify data and perform linear regression in mechanical and flow networks (64) as well as laboratory electrical networks (65). More generally, the mechanical behavior of epithelial tissues during development is extraordinary when viewed through the lens of ordinary passive materials. It remains to be seen how much of that behavior can be understood using “adaptive vertex models” (41) within a framework that replaces ordinarily fixed physical parameters with degrees of freedom that vary with time.

## Materials and Methods

Flies were maintained using standard methods, and embryos were collected and prepared for imaging and laser surgery as previously described (35, 66–68). Cell junctions were labeled via ubiquitous expression of DE-cadherin-GFP (69). Images were captured using Micro-Manager 2.0 software (Open Imaging) to operate a Zeiss Axiovert 200 M microscope outfitted with a Yokogawa CSU-W1 spinning disk confocal head (Solamere Technology Group), a Hamamatsu Orca Fusion BT camera, and a Zeiss 40X LD LCI PlanApochromat 1.2 NA multi-immersion objective (glycerin). Due to the embryo’s curvature, multiple z planes were imaged for each embryo at each time point to observe the dorsal opening. We recorded stacks with eight z-slices with 1 μm step size every 15 s throughout the closure duration, with a 100 ms exposure per slice.

Two-dimensional projections of the AS tissue were created from 3D stacks using DeepProjection (70). A custom Python algorithm was used to segment and track individual AS cells throughout dorsal closure (32): Briefly, binary masks of the AS cell boundaries and the amnioserosa tissue boundary (leading edge) were first predicted from microscopy movies using deep learning trained with expert-annotated dorsal closure specific data (71). Second, individual AS cells were segmented and tracked throughout the process using the watershed segmentation algorithm with propagated segmentation seeds from previous frames. Finally, for each cell, area, perimeter, aspect ratio and orientation in relation to the AS anterior-posterior axis were quantified over time. Based on the binary mask of the leading edge, we segmented the dorsal hole/AS shape, fitted an ellipse to it at each time point, and located the centroid position of each cell with respect to the long and short axis of the ellipse. This allowed us to precisely identify cells in the amnioserosa center (within 75% of the semi-major axis and 90% of the semi-minor axis), and exclude peripheral cells from comparisons between model and experiment. The straightness S of cell-cell junctions was quantified by segmenting the contour and end-to-end lengths of individual junctions using a graph-based algorithm (32).

Laser surgery was performed on a Zeiss Axio Imager M2m microscope equipped with a Yokogawa CSU-10 spinning disk confocal head (Perkin Elmer), a Hamamatsu EM-CCD camera and a Zeiss 40X, 1.2 NA water immersion objective. Micro-Manager 1.4.22 software (Open Imaging) controlled the microscope, the Nd:YAG UV laser minilite II (Continuum, 355 nm, 4 mJ, 1.0 MW peak power, 3–5 ns pulse duration, 10 Hz, (72)) and a steering mirror for laser incisions. In each embryo (N =48), 1 to 2 cuts of approx. 5 μm length with a laser setting at 1.4 μJ were performed in the bulk of the AS at different stages of closure (67, 73, 74) (Fig. S6A,B). The response of the AS was recorded prior to (~ 20 frames), during (~ 4 frames) and after (~ 576 frames) the cut at a frame rate of 5 Hz. The junction straightness S of each cut junction was manually quantified prior to the cut by manually tracing junction end-to-end length and junction contour length using ImageJ. Then, to analyze the initial recoil velocity, the motion of the vertices adjacent to the cut junction was followed in a kymograph perpendicular to the cut (line thickness 2 μm, Fig. S6A–C). On the basis of the kymograph, the distance d(t) between the two vertices of the severed junction was quantified manually over time using ImageJ. A double exponential function $a_{0}\text{ exp}\left(b_{0}t\right)+c_{0}\text{ exp}\left(d_{0}t\right)+e_{0}$ was fitted to d(t) (Fig. S6D). The initial slope of this function at t~0 corresponds to the initial recoil velocity $v_{r}$.

For the vertex model, we used the open-source CellGPU code (75). Analysis and illustration of model and experiment data was performed with custom Python scripts. Simulation code will be published on GitHub upon publication. Data associated with this study are available upon request.

## Supplementary Material

1

## Significance Statement

During embryogenesis, cells in tissues can undergo significant shape changes. Many epithelial tissues fluidize, i.e. cells exchange neighbors, when the average cell aspect ratio increases above a threshold value, consistent with the standard vertex model. During dorsal closure in *Drosophila melanogaster*, however, the amnioserosa tissue remains solid even as the average cell aspect ratio increases well above threshold. We introduce perimeter polydispersity and allow the preferred cell perimeters, usually held fixed in vertex models, to decrease linearly with time as seen experimentally. With these extensions to the standard vertex model, we capture experimental observations quantitatively. Our results demonstrate that vertex models can describe the behavior of the amnioserosa in dorsal closure by allowing normally fixed parameters to vary with time.
