# Supplementary material for: Minimal vertex model explains how the amnioserosa avoids fluidization during Drosophila dorsal closure
Source: ArXiv. 2023 Dec 20:arXiv:2312.12926v1. Preprint. [Version 1] (PMC10775355)
Supplement: 1 [file NIHPP2312.12926V1-supplement-1.pdf]

arXiv:2312.12926v1 [physics.bio-ph] 20 Dec 2023

Supplementary Material:
Minimal vertex model explains how the amnioserosa avoids fluidization during *Drosophila* dorsal closure

A. Short introduction to vertex models. Vertex models for tissues were motivated by two-dimensional models of dry foams made up of polygonal bubbles. In such foams, the vertices of bubbles tend to adjust their positions to balance forces arising from line tensions at the boundaries between adjacent bubbles; this force balance is achieved by minimizing the energy cost associated with bubble-bubble boundaries (1, 2). Tissue imaging typically provides time-evolving 2D projections of the tissue layers. 2D modeling of 2D projections in vertex models is appropriate, since tension is provided by a 1D ring of acto-myosin, and adhesion is provided by E-cadherin also in a ring-like geometry. One can visualize such a model as a tightly packed layer of elastic polygons, having a finite relaxed area, with a line tension around the circumference and "slippery" adhesiveness to their neighbors that prevents gaps, but allows sliding. With these assumptions, computer simulations show that barriers preventing cells from sliding past each other are low when cells are elongated, i.e., the tissue reacts like a fluid to external forces on time scales determined by the remaining friction. Barriers are high, in contrast, when cells are more rounded, i.e., the tissue reacts like a solid under external forces in the sense that the tissue may deform, but cells maintain their neighbors. This type of very simple modeling has been extraordinarily successful in providing quantitative predictions of the decrease and loss of solid character in a wide range of epithelial tissues (3–6) and monolayer cell cultures. It was found that one parameter, mean cell shape index ($\bar{q} = \langle p_i/\sqrt{a_i} \rangle$), where p is the cell perimeter and a is its area, was dominant in determining phase state in many systems (3–5). Within the standard vertex model, the AS is a two-dimensional plane comprising irregular polygons that represent cells and that display no overlaps or gaps, as shown in Fig. 1A,B in the main text. The positions of the vertices adjust to minimize the mechanical energy

$$E = \sum_{i=1}^{N} \frac{1}{2} k_a (a_i - a_0)^2 + \frac{1}{2} k_p (p_i - p_{0,i})^2. \qquad [1]$$

Here N is the total number of cells, p_i and a_i are the actual perimeter and area of cell i and $p_{0,i}$ and a_0 are its preferred perimeter and area. k_a and k_p represent the area and perimeter moduli of the cells. The first term in Eq. 1 represents the area elasticity of the cells. According to this term, there are restoring forces if the area of cell i, a_i, differs from its preferred area, a_0. We assume that the cell volumes are constant due to incompressibility, changes of area can arise from changes in the height of cells. We note that there are medioapical arrays of actomyosin that condense and relax as pulsing AS cells contract and expand (7). In the vertex model, the medioapical arrays would control the preferred area, $a_{0,i}$. In our model, we neglect such effects, taking $a_{0,i}$ to be constant in time and the same for every cell ($a_{0,i} = a_0 = \text{const}$).

The second term in Eq. 1, involving the cell perimeter, originates from the sum of two contributions

$$k_{p,i} p_i^2 + \gamma_i p_i = \frac{1}{2} k_p (p_i - p_{0,i})^2 \qquad [2]$$

where $p_{0,i} = -\gamma_i(2k_{p,i})$ is the effective target cell perimeter (10). The first term on the left side of Equ. 2 approximates active contractility of the actomyosin sub-cellular cortex. According to this term, the cortex acts like a spring that prefers each cell to have a perimeter of zero (complete contraction); this contributes a term proportional to the square of the perimeter (8). The second contribution (second term on left side of Eq. 2) arises from cell-cell adhesion and cortical tension which are proportional to the perimeter. They combine to give an effective line tension γ_i that penalizes a nonzero perimeter if $\gamma_i > 0$ (if contractility dominates) or rewards a nonzero perimeter if $\gamma_i < 0$ (if cell-cell adhesion dominates) (8–10).

B. Details of the amnioserosa vertex model. The initial AS cell sheet is modeled as a two-dimensional rectangular plane sheet with box size $L_x \times L_{y,\text{initial}}$ that is tiled with $N_{\text{initial}} = 256$ cells with no gaps or overlaps. We use periodic boundary conditions throughout the process. For simplicity, we set the preferred area for each cell i to be the same for all cells: $a_{0,i} = 1$.

The experimentally observed heterogeneity in cell shape index of the AS tissue is introduced in our model through the preferred perimeters of cells ($p_{0,i}$ for cell i). The initial values of $p_{0,i}$ are drawn from a Gaussian distribution with mean $\mu = 4.24$ and standard deviation $\sigma = 0.45$. We then minimize the energy using the FIRE algorithm (11) until the residual force on each vertex drops below 10^{-6} to obtain the initial state of the tissue. After minimization, we compare the distribution of shape index from both simulation and experiment (Fig. 1C).

We approximate the dorsal closure process by applying uni-axial quasi-static compression to our model tissue. We hold L_x fixed and with each step, we decrease L_y by $\epsilon = 0.01$ and re-equilibrate the system (minimize the mechanical energy) so that there is force balance at every vertex.

During the process, we also decrease all of the preferred cell perimeters with a linear rate corresponding to the average perimeter drop observed in the experiments ($m_p = 2.76$) (Fig. 1E):

$$p_{0,i}(\Delta A(t)) = p_{0,i}(\Delta A(0)) - m_p \Delta A(t). \qquad [3]$$

C. Relation between AS height and AS area. In Fig. 1 relation between the AS height and area is demonstrated.

D. Cell shape index and aspect ratio. The shape index of cell i is defined as $q_i \equiv p_i/\sqrt{a_i}$, where p_i is the cell perimeter and a_i is the cell area. The mean cell shape index is $(\bar{q}) = \langle \frac{p_i}{\sqrt{a_i}} \rangle = \frac{1}{N}\sum_{i=1}^{N} \frac{p_i}{\sqrt{a_i}}$.

The aspect ratio of a cell is defined as

$$\alpha = \frac{\sqrt{\frac{1}{\lambda_1}}}{\sqrt{\frac{1}{\lambda_2}}} \quad [4]$$

where λ_1 and λ_2 are smallest and largest eigenvalues of the shape tensor I, defined as

$$I = \begin{pmatrix} I_{XX} & I_{XY} \\ I_{XY} & I_{YY} \end{pmatrix} \quad [5]$$

Here, $I_{XX} = \iint_R y^2 dA$, $I_{YY} = \iint_R x^2 dA$, and $I_{XY} = \iint_R xy dA$. The tensor I, calculated as in Ref. (4), corresponds to the moment-of-inertia tensor in the case where the mass density per unit area is constant. Thus I measures the second area moment of the polygon representing the apical surface of the cell, relative to a fixed point, weighting each infinitesimal area element of the cell equally. In this case, the fixed point is the center of the cell, as defined by the first moment (the mean) of the area distribution of the polygon. Thus, the second moment measures the variance of the area distribution. In 2-dimensions, the tensor is a 2×2 matrix as defined above and its two eigenvalues λ_1 and λ_2 along two directions that in the case of an ellipse would correspond to the minor and major axes, respectively.

E. Cell orientational order parameter. During dorsal closure, cells tend to align along the anterior-posterior axis. According to the standard vertex model, this alignment should enhance the solid character of the tissue (5). To better understand the relationship of solidity to the collective alignment among aminoserosa cells, we quantify their degree of orientational order (12–14). This tells us the degree to which cells align relative to the anterior-posterior axis. The 2D orientational order parameter $\bar{Q}$ is (15)

$$\bar{Q} = \frac{1}{N}\sum_{i=1}^{N} \cos(2\theta_i) \quad [6]$$

where θ is the angle of the major axis of the cell relative to the anterior-posterior axis. As described in Sec D, the direction of the cell's major axis is given by the largest eigenvector of the shape tensor I. Note $\bar{Q} = 0$ when cells are randomly aligned in the tissue (the isotropic case) and $\bar{Q} = 1$ if all cells are perfectly aligned along the anterior-posterior axis.

F. Relation between mean and standard deviation of cell aspect ratio. Similar to the cell shape index q, the cellular aspect ratio α also characterizes cell shape (4). Fig. 2 demonstrates that our model successfully captures experimental observations for both the mean and standard deviations of the tissue aspect ratio.

G. Rigidity percolation in uniaxially deformed tissue. Uniaxial deformation during dorsal closure produces anisotropy in the tissue which can affect the percolation threshold. To probe how the percolation threshold f_c depends on the uniaxial deformation, we take the model tissue at each value of $\Delta A(t)$ and randomly assign each cell-cell junction to have nonzero tension with probability f (Fig. 3A). Percolation of the system as a whole requires percolation in both the x and y direction. Therefore, the percolation threshold of the system is given by $f_c = \max[f_c^x, f_c^y]$ (Fig. 3C,D). To determine the percolation thresholds f_c^x and f_c^y at different stages of closure ΔA, we each assessed the probability of obtaining a system-spanning connected path of non-zero tension edges $P_{tension}^{X,Y}$ for different ratios of junctions with non-zero tension f_r (Fig. 3C,D). The respective percolation thresholds $(f_c) : (\frac{d^2 P_{tension}}{df_r^2} = 0)$ were the inflection points of $P_{tension}$. In Fig. 3B, note that $f_c^x \approx 0.66$ remains unchanged but f_c^y decreases with increasing $\Delta A(t)$. As a result, $f_c = \max[f_c^x, f_c^y]$ remains constant at $f_c \approx 0.66$ throughout dorsal closure. Thus we find that the fraction of junctions with non-zero tension must satisfy $f_r \geq f_c \approx 0.66$ for solid behavior.

Uniaxial deformation can also affect f_r, the fraction of junctions with non-zero tension. To determine the fraction of junctions with non-zero tension in the model, we chose a threshold corresponding to the noise floor of the tension (16), so that junctions with $\tau_J > 10^{-4}$ are counted as having finite tension. In the experiments, we found that the initial recoil velocity v_r was independent of junction straightness S for $S \lesssim 0.93$ (Fig. 2E), but rose linearly with S above this threshold. We therefore assume that only junctions with $S > 0.93$ carry tension and contribute to solid response. In Fig. 3B it is evident that $f_r > f_c$ throughout the process, consistent with the system being solid.

In summary, the AS tissue maintains a percolating network of tense cell-cell junctions across the dorsal opening during the entire process of closure, consistent with its solid character.

The decrease of preferred cell perimeter is crucial. If we leave the preferred perimeter fixed at its initial value in our model, we obtain f_r as shown in Fig. 3B. Clearly, f_r falls below f_c so that the system fluidizes.

H. Mean junction tension in tissues with and without orientational alignment of the cells. To understand the effect of the orientational alignment of cells on mechanical stiffness in our model, we compared our model, where we found orientational alignment of cells (Fig. 2C) during uni-axial constriction, with simulations with randomly aligned cells. We then quantified the mean junction tension $\bar{\tau}_J$, as a measure for tissue stiffness, in model configurations with different mean shape indices $\bar{q}$. For a given mean shape index, we find that the junction tension is higher if the cells are oriented than if they are not (Fig. 4), consistent with Ref. (5).

I. Effect of uniaxial deformation on tissue phase state. We studied the effect of uniaxial deformation of the AS, as implemented in our model, in contrast to isotropic deformation. We progressively decreased the tissue size in our model in both x and y direction, and compared it with our model results for uniaxial deformation at each given ΔA in terms of mean shape index $\bar{q}$ (Fig. 5A), average orientational order $\bar{Q}$ (Fig. 5B), average junction tension $\bar{\tau}_J$ and initial recoil velocity v_J (Fig. 5C), and standard deviation of shape index σ_q (Fig. 5D). We found that uniaxial deformation of the AS in our is crucial to recapitulate the experimentally measured time courses of cell-shape features.

To test the effect of uniaxial vs. isotropic deformation on the solid phase of the AS, we assessed the fraction of junctions with nonzero tension f_r during closure (red dashed line in Fig. 3B). For isotropic deformation, our model predicts f_r well above the percolation threshold f_c, so non-zero tension junctions percolate in either case (isotropic and uni-axial).

1. D Weaire, N Rivier, Soap, cells and statistics—random patterns in two dimensions. *Contemp. Phys.* **25**, 59–99 (1984).
2. F Graner, Y Jiang, E Janiaud, C Flament, Equilibrium states and ground state of two-dimensional fluid foams. *Phys. Rev. E* **63**, 011402 (2000).
3. JA Park, et al., Unjamming and cell shape in the asthmatic airway epithelium. *Nat Mater* **14**, 1040–1048 (2015).
4. L Atia, et al., Geometric constraints during epithelial jamming. *Nat. Phys.* **14**, 613–620 (2018).
5. X Wang, et al., Anisotropy links cell shapes to tissue flow during convergent extension. *Proc. Natl. Acad. Sci.* **117**, 13541–13551 (2020).
6. J Devany, DM Sussman, T Yamamoto, ML Manning, ML Gardel, Cell cycle–dependent active stress drives epithelia remodeling. *Proc. Natl. Acad. Sci.* **118**, e1917853118 (2021).
7. RP Moore, et al., Superresolution microscopy reveals actomyosin dynamics in medioapical arrays. *Mol. Biol. Cell* **33**, ar94 (2022) PMID: 35544300.
8. R Farhadifar, JC Röper, B Aigouy, S Eaton, F Jülicher, The influence of cell mechanics, cell-cell interactions, and proliferation on epithelial packing. *Curr. biology: CB* **17**, 2095–2104 (2007).
9. GW Brodland, The Differential Interfacial Tension Hypothesis (DITH): a comprehensive theory for the self-rearrangement of embryonic cells and tissues. *J. Biomech. Eng.* **124**, 188–197 (2002).
10. D Bi, JH Lopez, JM Schwarz, ML Manning, A density-independent rigidity transition in biological tissues. *Nat. Phys.* **11**, 1074–1079 (2015).
11. E Bitzek, P Koskinen, F Gähler, M Moseler, P Gumbsch, Structural relaxation made simple. *Phys. Rev. Lett.* **97**, 170201 (2006).
12. P de Gennes, J Prost, *The Physics of Liquid Crystals*, International series of monographs on physics. (Clarendon Press), (1993).
13. G Duclos, S Garcia, HG Yevick, P Silberzan, Perfect nematic order in confined monolayers of spindle-shaped cells. *Soft Matter* **10**, 2346–2353 (2014).
14. X Li, et al., On the mechanism of long-range orientational order of fibroblasts. *Proc. Natl. Acad. Sci.* **114**, 8974–8979 (2017).
15. PG de Gennes, Possible experiments on two-dimensional nematics. *Symp. Faraday Soc.* **5**, 16–25 (1971).
16. X Li, A Das, D Bi, Mechanical heterogeneity in tissues promotes rigidity and controls cellular invasion. *Phys. Rev. Lett.* **123**, 058101 (2019).

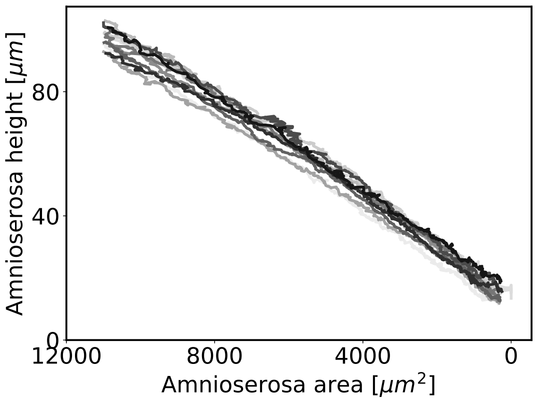

Fig. 1. Linear relation between the AS height and area. Each color represents one embryo (N=12).

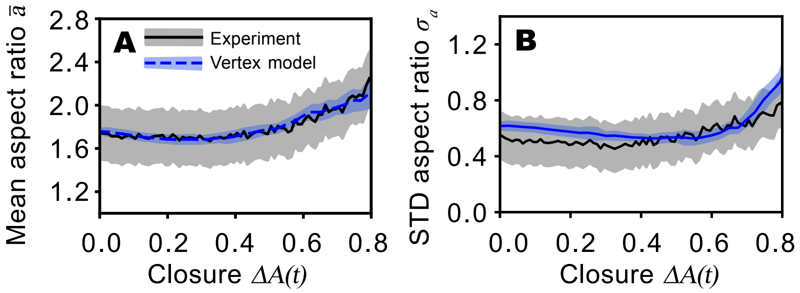

Fig. 2. Comparison of mean (A) and standard deviation (B) of cell aspect ratio between experimental data (black) and model (blue) during dorsal closure. Lines show mean, and shaded areas standard deviation of various initial configurations (model, N = 10) or different embryos (experiment, N = 12).

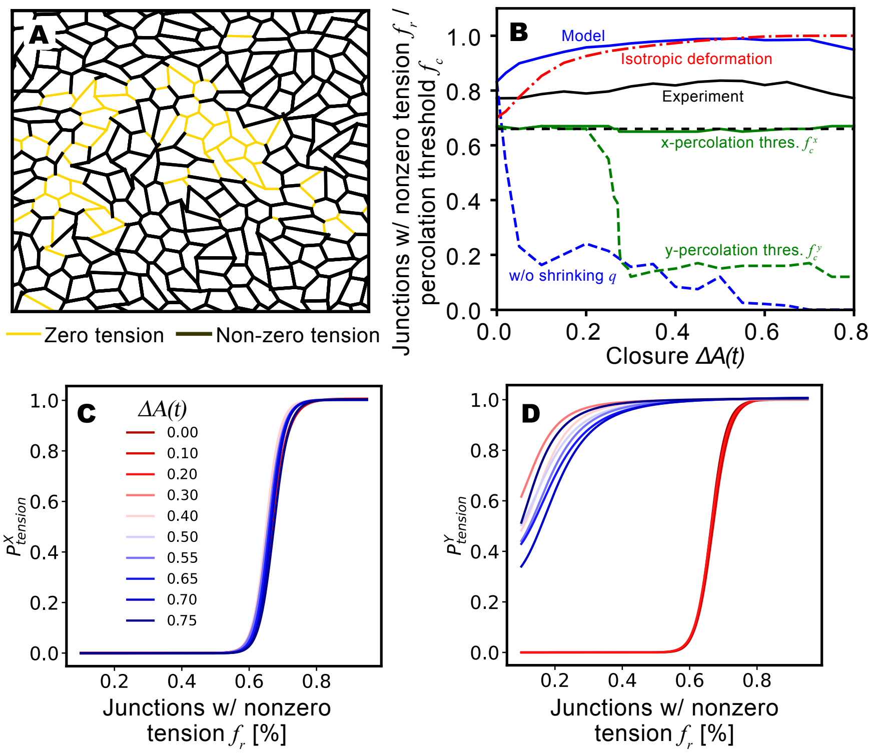

Fig. 3. Analysis of percolation in junction network. (**A**) Snapshot of tension network percolation: Black thick lines are edges with finite tensions (percolating throughout the tissue), while other edges have $\tau = 0$. (**B**) Fraction of junctions with nonzero tension, f_r, in experiment (black solid) and model (blue solid) as a function of $\Delta A(t)$. The model predicts the minimum fraction f_c needed for percolation of junctions with nonzero tension in the x (green solid) and y (green dashed) directions. In both experiment and model, $f_r > f_c = \max[f_c^x, f_c^y]$, indicating that the system is solid. Black dashed line shows the effective bond percolation threshold $f_c^* = 0.66 = \max[f_c^x, f_c^y]$ above which the system is solid. If cell perimeters, and accordingly the shape indices of cells are held constant instead of shrinking with increasing $\Delta A(t)$, our model predicts that f_r drops below the percolation threshold (blue dashed), leading to fluidization. If we apply isotropic instead of uni-axial deformation, f_r (red dashed) remains above the percolation threshold. (**C**) Probability of obtaining percolation of cell edges with nonzero tensions ($P^X_{tension}$) vs. f_r in the X directions (left) of our model tissue, at various closure stages (legend), which correspond to various tissue anisotropy stages. The point of inflection is the respective percolation threshold f_c. (**D**) Same plot as **C** for Y direction, showing substantially lower percolation thresholds than for X direction at late closure stages.

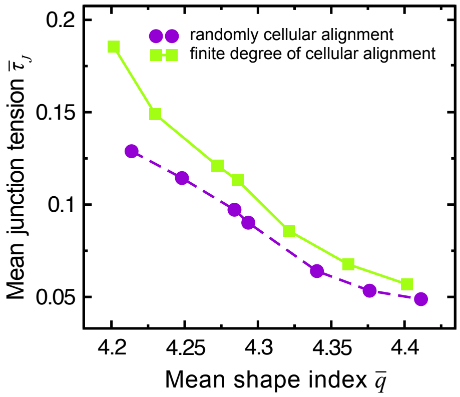

Fig. 4. Comparison of mean junction tension $\bar{\tau}_J$ for tissues with and without cellular orientational alignment at different mean cell shape indices $\bar{q}$.

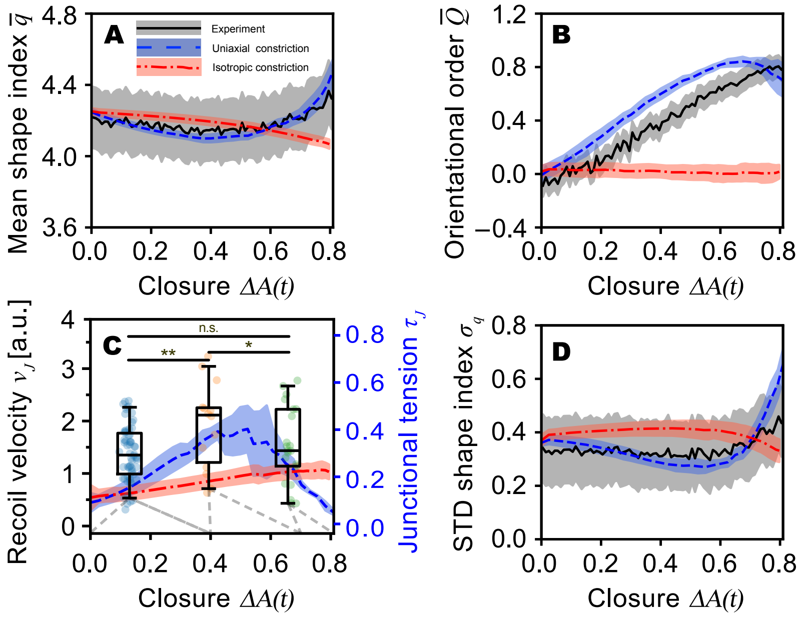

Fig. 5. Comparison of experimental data (black) with model results with uniaxial deformation (blue) and isotropic deformation (red) during dorsal closure. (A) Time course of mean shape index $\bar{q}$. **(B)** Time course of average orientational order parameter $\bar{Q}$. **(C)** Box plot of initial junction recoil velocity of junction vertices after performing laser ablation of the junction, and time-course of average junction tension $\bar{\tau_c}$. Boxplots show data for three intervals of ΔA ($\Delta A < 0.4$, $0.4 \leq \Delta A < 0.7$, $\Delta A \geq 0.7$). Whiskers extend to the 5th and 95th percentiles, while the boxes delineate the interquartile range, and the horizontal lines within the boxes indicate the median values. An ANOVA followed by a post-hoc Tukey's HSD test was conducted to assess statistical significance (*: $p < 0.1$, **: $p < 0.05$). We performed and evaluated cuts of $N = 97$ junctions. **(D)** Time-course of standard deviation of shape index (σ_q). Lines show mean, and equally color intervals show respective standard deviations (N = 10 model initial configurations, N = 12 embryos).

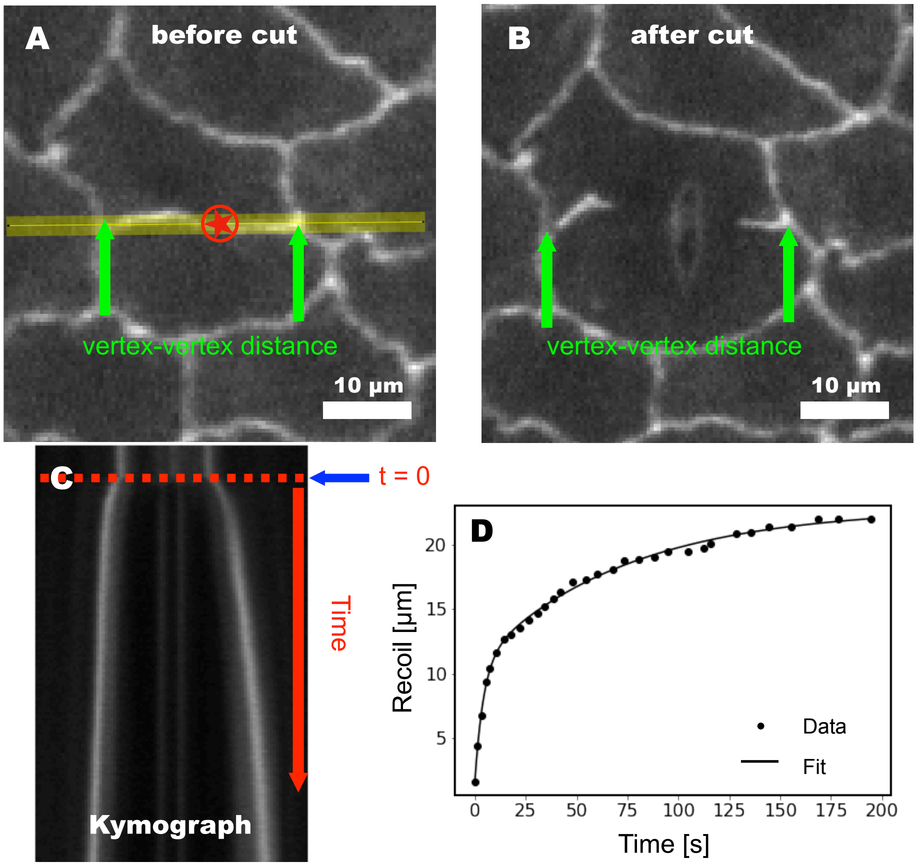

Fig. 6. Measurement and evaluation of initial recoil velocity of vertices after laser cut. (**A**),(**B**) Confocal fluorescent images of AS cells. The red star in (A) marks the ablation region. Green arrows show the vertex positions just prior to (A) and after (B) ablation. The yellow line (A) along which the motion of vertices is assessed using kymograph analysis. (**C**) Kymograph of positions of the two vertices showing recoil after ablation (the red dotted line shows time of cut at $t = 0$). (**D**) Time-course of recoil after ablation. The recoil was well fitted by a double exponential function $a_0 \exp(b_0 t) + c_0 \exp(d_0 t) + e_0$.
